# *T*max volume can predict clinical type in patients with acute ischemic stroke

**DOI:** 10.1002/brb3.3163

**Published:** 2023-07-20

**Authors:** Fumihisa Kishi, Ichiro Nakagawa, Seigo Kimura, Daiji Ogawa, Ryokichi Yagi, Keiichi Yamada, Hirokatsu Taniguchi

**Affiliations:** ^1^ Department of Neurosurgery Yagi Neurosurgical Hospital Higashinariku Osaka Japan; ^2^ Department of Neurosurgery Nara Medical University Kashihara Nara Japan; ^3^ Department of Neurosurgery Osaka Medical and Pharmaceutical University Takatsuki Osaka Japan

**Keywords:** acute ischemic stroke, atherosclerosis, CT perfusion, mechanical thrombectomy

## Abstract

**Objective:**

Endovascular therapy (EVT) is performed for acute ischemic stroke (AIS) with large vessel occlusion (LVO), however, the treatment strategies and clinical outcomes differ between cardiac embolism (CE) and intracranial arteriosclerosis‐derived LVO (ICAS‐LVO). We analyzed whether the time‐to‐max (*T*max) volume derived from perfusion imaging predicted clinical classification in AIS patients before EVT.

**Methods:**

Consecutive AIS patients with anterior circulation LVO evaluated by automated imaging software were retrospectively identified. Patients were classified into a CE group and an ICAS‐LVO group, and parameters were compared between groups.

**Results:**

Thirty‐nine patients were included and *T*max volume and *T*max > 6 s volume/*T*max > 4 s volume were significantly greater in the CE group than in the ICAS‐LVO group (*T*max > 4 s volume: 261 mL vs. 149 mL, *p* = .01, *T*max > 6 s volume: 143 mL vs. 59 mL, *p* = .001, *T*max > 6 s volume/*T*max > 4 s volume: 0.59 vs. 0.40, *p* < .001). Multiple logistic regression analysis indicated an association between clinical classification and high *T*max > 6 s volume/*T*max > 4 s volume (*p* = .04).

**Conclusion:**

The *T*max volume derived from perfusion imaging predicts the clinical classification of AIS patients before EVT.

## Graphical abstract

Acute ischemic stroke (AIS) due to large vessel occlusion (LVO) is indicated for endovascular treatment (EVT), but treatment strategies and clinical outcomes differ depending on clinical type between cardiac embolism‐induced cerebral LVO and intracranial atherosclerosis‐related LVO. The present study showed that *T*max volumes and *T*max > 6 s volume/*T*max > 4 s volume obtained from perfusion imaging can predict the clinical type in patients with AIS prior to EVT.

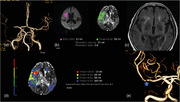

## 1 INTRODUCTION

Acute ischemic stroke (AIS) due to cerebral large vessel occlusion (LVO) has become an indication for endovascular treatment (EVT), and eligibility has expanded in recent years (Albers et al., 2018; Nogueira et al., 2018). Cerebral LVO is caused by various clinical types, including cardiac embolism (CE), atherothrombotic infarction, and artery‐to‐artery embolism (Lee et al., 2018). Knowledge of the clinical type before EVT is obviously desirable. In particular, differentiation between CE‐induced cerebral LVO (CE‐LVO) and intracranial atherosclerosis‐related LVO (ICAS‐LVO) is important in developing a therapeutic strategy for EVT. However, the identification of clinical type before EVT remains challenging in patients with complicated clinical situations. We introduced fully automated image processing software (Rapid Processing of Perfusion and Diffusion; RAPID) in October 2020, and actively performed CT perfusion (CTP) and MRI perfusion (MRP) even for cases within 6 h of onset. In evaluating the analysis by RAPID, time‐to‐maximum (*T*max) volume seemed to show different characteristics according to clinical type. *T*max volume is often used to estimate penumbra tissue (Albers, 2018; Inoue, 2019). *T*max volumes have been reported in association with collateral flow or clinical outcomes (Arenillas et al., 2018; Fainardi et al., 2022; Galinovic et al., 2016; Lyndon et al., 2021), but few studies have reported on the association between *T*max volume and clinical type, particularly in terms of differentiating between ICAS‐LVO and CE LVO (Haussen et al., 2018).

Accurate prediction of the clinical type of AIS prior to EVT can accurately determine not only the indication for EVT, but also the timing of antiplatelet administration and the choice of device for EVT. The aim of the present study was to evaluate whether *T*max volume can predict clinical type in patients with AIS prior to EVT.

## 2 MATERIALS AND METHODS

This retrospective, observational study was approved by the institutional review board of our university hospital (approval no. 3138) and was performed according to the guidelines of the Strengthening the Reporting of Observational Studies in Epidemiology statement. All patients were provided with a document explaining CT or MRI examination. Written informed consent was obtained from each patient or from their legally authorized representatives at admission.

### 2.1 Patient selection

We identified consecutive patients with AIS who met the following eligibility criteria: (1) presentation to our hospital due to AIS between October 2020 and October 2022; (2) presentation with middle cerebral artery (MCA) M2 segment, M1 segment, or internal carotid artery (ICA) occlusion; (3) perfusion imaging within 24 h of the last‐known‐well time or with unknown onset time; (4) diagnosis of CE or ICAS‐LVO. We excluded patients who: (1) presented with no cerebral artery occlusion; (2) presented due to LVO with another cause, such as artery‐to‐artery embolism or dissection; (3) presented with occlusion of the MCA M3 segment, anterior cerebral artery, or posterior circulation, or chronic occlusion; or (4) presented with poor perfusion imaging.

### 2.2 Image analysis

Perfusion imaging was conducted using a 3.0‐T MR system (Signa HDx^®^; GE Healthcare) or a 64‐row detector CT system (Revolution Maxima^®^; GE Healthcare). Either MRP or CTP was taken at the discretion of the doctor. A single‐shot spin‐echo EPI‐DWI sequence was used with the following parameters: *b*‐values, 1000 s/mm^2^; slice thickness, 5 mm. MRP images were acquired with a gradient‐echo echo‐planar technique. Gadoteridol was power‐injected through a peripheral intravenous catheter at doses standardized by patient body weight (0.2 mL/kg body weight, to a maximum of 20 mL) at 2–4 mL/s, immediately followed by a 20‐mL saline flush at the same rate. CTP was performed two times at the parietal and basal sides. Forty milliliters of iopamidol (370 mg iodine/mL) was power‐injected through a peripheral intravenous catheter at 4–6 mL/s at one time and immediately followed by a 20‐mL saline flush at the same rate. Estimates of the volume of the ischemic core and penumbral regions from CTP or MRI diffusion and perfusion scans were calculated with the use of RAPID software. Ischemic core volumes were based on a RAPID relative cerebral blood flow (CBF) lesion volume using a ≺30% threshold or a RAPID DWI lesion volume with an apparent diffusion coefficient (ADC) threshold of <620 × 10^−3^ mm^2^/s. The volume of penumbral volumes was estimated using the *T*max perfusion parameter with a threshold of >6 s (*T*max > 6 s) on both CTP and MRP (Albers et al., 2018). Incorrect estimation of the core regions by RAPID was manually reanalyzed and cases with poor images due to motion artifacts or low cardiac output were excluded (Laughlin et al., 2019 ). Cases were then classified to a CE group or ICAS‐LVO group. Clinical type was diagnosed by imaging (cerebral angiography, MRI/MR angiography, and CT/CT angiography). The ICAS‐LVO group included only cases in which it was clear that intracranial atherosclerosis led to acute occlusion. If imaging had been performed before onset, those images were used for clinical type diagnosis. For cases in which EVT was performed, the properties of the thrombus and the presence of intracranial arterial stenosis after EVT were also used for the diagnosis of clinical type.　For example, ICAS‐LVO was diagnosed when there was stenosis in the pathological vessels on imaging before onset or after EVT and white thrombus was retrieved. Two board‐certified radiologists with over 15 years of experience in neuroimaging who were blinded to the clinical details reviewed all images and conducted the measurements.

### 2.3 Outcome measures

The following values were measured by analysis of RAPID: DWI‐perfusion‐weighted imaging (PWI) mismatch, CBF‐PWI mismatch, core volume, mismatch volume, *T*max > 10 s volume, *T*max > 8 s volume, *T*max > 6 s volume, *T*max > 4 s volume, *T*max > 10 s volume/*T*max 6 s volume (hypoperfusion intensity ratio [HIR]), *T*max > 8 s volume/*T*max > 4 s volume, and *T*max > 6 s volume/*T*max > 4 s volume. In addition, the collateral score was measured in cases in which cerebral angiography was performed (Higashida et al., 2003), Comparisons between patients in the CE and ICAS‐LVO groups were performed for the variables shown in Tables 1 and  2.

**TABLE 1:** Baseline characteristics between CE and ICAS‐LVO.

	Total	CE	ICAS‐LVO	
Characteristics	N = 52	N = 40	N = 12	*p* Value
Age, median, years (IQR)	83 (76–87)	84 (77–88)	80 (68–83)	.08
Male, n (%)	25 (48)	19 (48)	6 (50)	1.00
Pre‐mRS (0–2), n (%)	31 (60)	20 (50)	11 (92)	.017
Median arrival NIHSS (IQR)	15.5 (7.0–20.0)	17.5 (12.8–20.3)	3.5 (3.5–7.0)	<.001
Median systolic blood pressure, mmHg (IQR)	155 (141–165)	155 (137–165)	150 (142–163)	.84
Atrial fibrillation, n (%)	35 (67)	34 (85)	1 (8)	<.001
INR, median (IQR)	0.97 (0.90–1.03)	1.00 (0.90–1.03)	0.95 (0.90–1.03)	.67
BS, median, mg/dL (IQR)	118 (106–147)	117 (109–141)	137 (100–189)	.49
HbA1c, median, % (IQR)	5.60 (5.40–6.00)	5.60 (5.40–5.90)	6.05 (5.58–7.23)	.02
BNP, median, pg/mL (IQR)	166 (61–275)	211 (84–428)	62 (16–100)	.007
D‐dimer, median, μg/mL (IQR)	2.0 (1.3–4.8)	2.2 (1.5–5.5)	1.5 (0.8–2.1)	.04
LDL‐cho, median, mg/dL (IQR)	101 (84–128)	99 (83–123)	127 (97–182)	.07
Median LKW time to perfusion image time, min (IQR)	181 (72–389)	131 (65–276)	611 (213–688)	.009
Side rt, n (%)	25 (48)	17 (43)	8 (67)	.19
Location				.51
M1, n (%)	26 (50)	19 (48)	7 (58)	
M2, n (%)	10 (19)	7 (18)	3 (25)	
IC, n (%)	16 (31)	14 (35)	2 (16)	
Median DWI‐ASPECTS (IQR)	7.5 (5.8–9.0)	7.0 (5.0–8.0)	9.5 (8.0–10.0)	.001
rtPA, n (%)	15 (29)	14 (35)	1 (8)	.14
EVT, n (%)	35 (67)	29 (73)	6 (50)	.056
90 days mRS (0–2), n (%)	18 (35)	11 (28)	7 (58)	.08

BS, blood sugar level; DWI‐ASPECTS, Diffusion Weighted Imaging‐Alberta Stroke Program Early CT Score; EVT, endovascular treatment; IC, internal carotid artery; LKW time, last known well time; M1, middle cerebral artery M1 segment; mRS, modified Rankin Scale; NIHSS, National Institutes of Health Stroke Scale; rtPA, recombinant tissue‐type plasminogen activator.

**TABLE 2:** Outcomes of perfusion imaging between CE and ICAS‐LVO.

	Total	CE	ICAS‐LVO	
Characteristics	N = 52	N = 40	N = 12	*p* Value
DWI‐PWI/CBF‐PWI mismatch positive, n (%)	43 (83)	33 (83)	10 (83)	1.00
Core volume (ADC < 620 × 10^−6^ mm^2^/s), median, mL (IQR)	15 (0–58)	34 (0–87)	0 (0–14)	.008
*T*max > 4 s volume, median, mL (IQR)	234 (140–298)	261 (164–323)	149 (91–221)	.01
*T*max > 6 s volume, median, mL (IQR)	131 (57–182)	143 (87–190)	59 (24–95)	.001
*T*max > 8 s volume, median, mL (IQR)	72 (22–129)	92 (52–141)	15 (11–23)	<.001
*T*max > 10 s volume, median, mL (IQR)	43 (12–99)	58 (30–109)	8 (0–11)	<.001
*T*max > 10 s volume/*T*max > 6 s volume (HIR), median (IQR)	0.41 (0.24–0.60)	0.50 (0.33–0.64)	0.13 (0–0.29)	<.001
*T*max > 6 s volume/*T*max > 4 s volume, median (IQR)	0.53 (0.42–0.68)	0.59 (0.50–0.70)	0.40 (0.30–0.45)	<.001
*T*max > 8 s volume/*T*max > 4 s volume, median (IQR)	0.35 (0.17–0.48)	0.38 (0.31–0.53)	0.11 (0.09–0.18)	<.001
Conventional angiographic collateral score	N = 38	N = 29	N = 9	.007
0–1, n (%)	20 (53)	19 (66)	1 (11)	
2–3, n (%)	15 (40)	10 (35)	5 (56)	
4, n (%)	3 (10)	0 (0)	3 (43)	

CBF, cerebral blood flow; DWI, diffusion‐weighted imaging; HIR, hypoperfusion intensity ratio; PWI, perfusion‐weighted imaging; *T*max, time‐to‐maximum.

### 2.4 Statistical analysis

The descriptive statistics of baseline variables were calculated and are reported as the median and interquartile range (IQR) for non‐normal or ordinal data and as proportions for binary data. Fisher's exact test was used for the comparison of categorical variables, and the Mann–Whitney U test or Kruskal–Wallis test was used to compare continuous variables. To evaluate the strength of the *T*max parameter to predict clinical type, receiver operating characteristic curves were constructed that plotted sensitivity against 100 minus specificity. The optimum cutoff values for the best combination of sensitivity and specificity were then calculated. Multivariable logistic regression analysis was applied to examine the independent associations of *T*max parameters with clinical type. Explanatory variables were chosen if *p*‐values in univariate analysis for clinical type were statistically significant. Statistical significance was set at the level of *p* < .05. All statistical analyses were performed using the EZR statistical software package (version 1.41) (Kanda, 2013).

## 3 RESULTS

**FIGURE 1:**
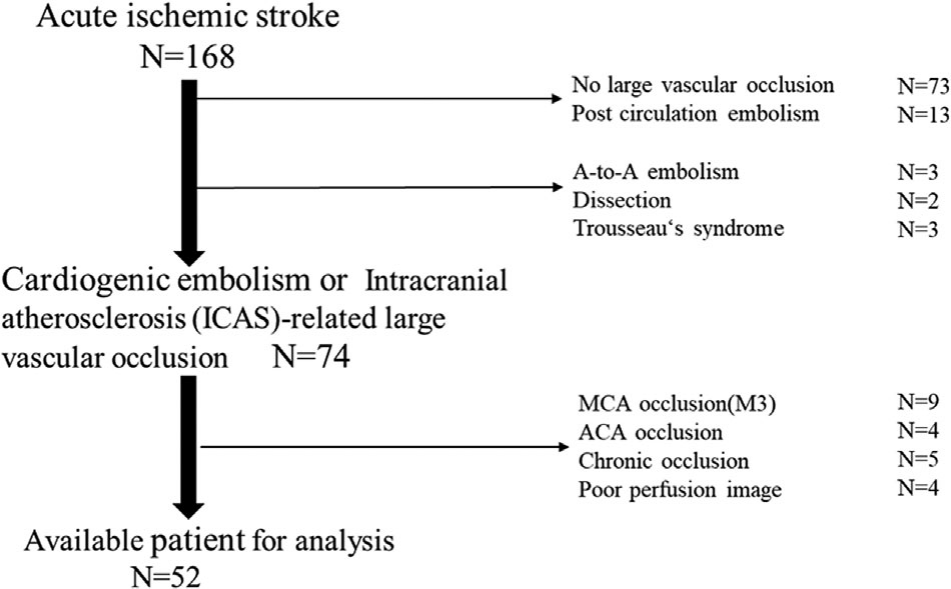
Patient selection. MCA: middle cerebral artery, ACA: anterior cerebral artery.

During the study period, 168 patients underwent perfusion imaging and analysis using RAPID. Of these, 116 patients were excluded for the following reasons: no cerebral artery occlusion (n = 73), posterior circulation occlusion (n = 13), main artery occlusion due to another cause (n = 8), MCA M3 segment (n = 9), anterior cerebral artery (n = 4), or chronic occlusion (n = 5), and poor perfusion imaging (n = 4). Fifty‐two patients (27 females) were included in the analysis (Figure 1), with 40 patients in the CE group and 12 patients in the ICAS‐LVO group. Median age was 83 years (IQR, 76−87 years). Twenty‐one patients (60%) showed prestroke mRS 0−2 and 35 patients (67%) had AF. Median NIHSS on admission was 15.5 (IQR, 7.0−20.0). Twenty four patients received CTP, and 28 patients received MRP (Table 1). DWI‐PWI mismatch/CBF‐PWI mismatch was observed in 43 of the 52 patients (83%). Median core volume was 15 mL (IQR, 0–58), median *T*max > 6 s volume was 131 mL (IQR, 57–182), median *T*max > 6 s volume/*T*max > 4 s volume was 0.53 (IQR, 0.42−0.68) (Table 2).

**FIGURE 2:**
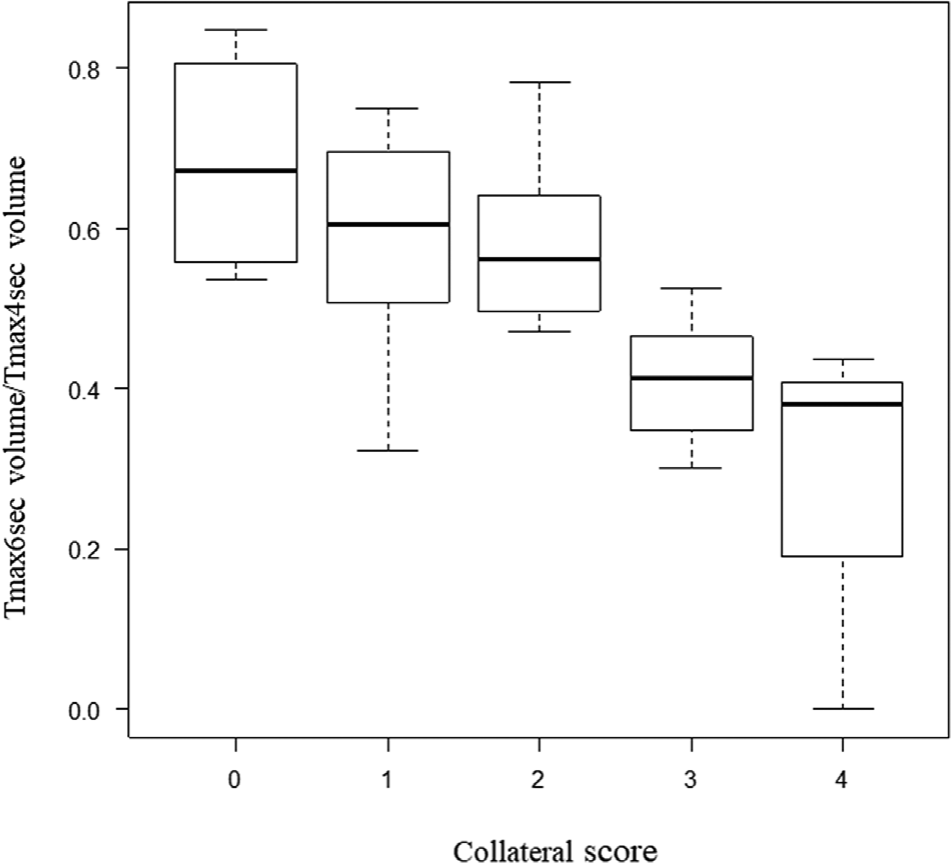
Outcomes of *T*max > 6 s volume/*T*max > 4 s volume for collateral score.

The results of univariate analysis were as follows: Patients in the CE group showed a higher median NIHSS on arrival, a higher frequency of AF, a lower median HbA1c, a higher median BNP, a higher median D‐dimer, a shorter median last‐known‐well time to perfusion imaging time, and a lower median DWI‐ASPECTS than patients in the ICAS‐LVO group (Table 1). Patients in the CE group had a larger median core volume, *T*max volume, and *T*max ratio than patients in the ICAS‐LVO group (Table 2). Patients in the CE group had larger median *T*max > 6 s volume/*T*max > 4 s volume than patients in the ICAS‐LVO group for every location of occlusion, although there was no significant difference (Table 3). In thirty‐eight patients who received cerebral angiography or EVT, patients in the ICAS‐LVO group were more likely to have intermediate or excellent collateral flow, whereas those of CE group were more likely to have poor collateral flow (*p* = .007) (Table 2). Although no significant difference was observed between every collateral score group due to the small number of cases, the higher the collateral score, the lower the *T*max > 6 s volume/*T*max > 4 s volume in the Kruskal–Wallis test (*p* = .003) (Figure 2).

**TABLE 3:** **Outcomes of *T*max > 6 s volume/*T*max > 4 s volume between CE and ICAS‐LVO for every location of occlusion**.

	CE		ICAS‐LVO		
	N	Median *T*max > 6 s volume/*T*max > 4 s volume (IQR)	N	Median *T*max > 6 s volume/*T*max > 4 s volume (IQR)	*p*‐Value
Location					
IC	14	0.61 (0.46–0.69)	2	0.36 (0.32–0.40)	.10
M1	19	0.54 (0.51–0.69)	7	0.42 (0.36–0.49)	.01
M2	7	0.59 (0.45–0.70)	3	0.36 (0.29–0.37)	.27

IC, internal carotid artery; M1, middle cerebral artery M1 segment; M2, middle cerebral artery M2 segment; *T*max, time‐to‐maximum.

**FIGURE 3:**
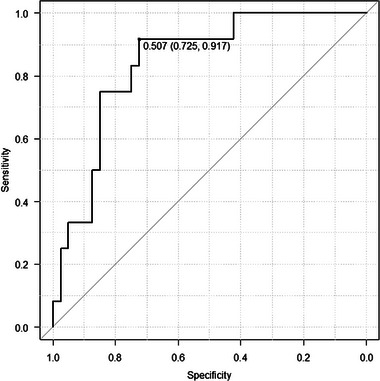
ROC curve analysis.

The critical *T*max > 6 s volume/*T*max > 4 s volume cutoff value for predicting clinical diagnosis was determined to be 0.507 using receiver operating characteristic curve analysis, yielding optimal sensitivity of 0.725, specificity of 0.917, positive predictive value of 0.935, negative predictive value of 0.476, area under the curve of 0.84, and 95% confidence interval of 0.725–0.959. (Figure 3) Accordingly, *T*max > 6 s volume/Tmax > 4 s volume was classified as high (>0.5) or low (≤0.5). For multiple logistic regression analysis, variables with high *T*max > 6 s volume/*T*max > 4 s volume and a significant difference of *p* < .01 in univariate analysis were selected as explanatory variables. Multiple logistic regression analysis showed that high *T*max > 6 s volume/*T*max > 4 s volume was associated with clinical type (odds ratio, 13.0; 95% CI, 1.13–150; *p* = .04) (Table 4).

**TABLE 4:** Logistic regression analysis of clinical and imaging features.

	Odds ratio	95% CI	*p*‐Value
AF BNP NIHSS LKW time to perfusion image time,	0.32 1.00 0.90 1.00	0.03–3.09 0.995–1.00 0.77–1.07 0.998–1.00	.33 .91 .24 .65
DWI‐ASPECTS Core volume	1.41 0.97	0.70–2.85 0.93–1.02	.33 .27
High *T*max > 6 s volume/*T*max > 4 s volume	13.0	1.13–150	.04

AF, atrial fibrillation; BNP, brain natriuretic peptide; DWI‐ASPECTS, Diffusion Weighted Imaging‐Alberta Stroke Program Early CT Score; LKW time, last known well time; NIHSS, National Institutes of Health Stroke Scale.

### 3.1 Case presentation

#### 3.1.1 Case 1

**FIGURE 4:**
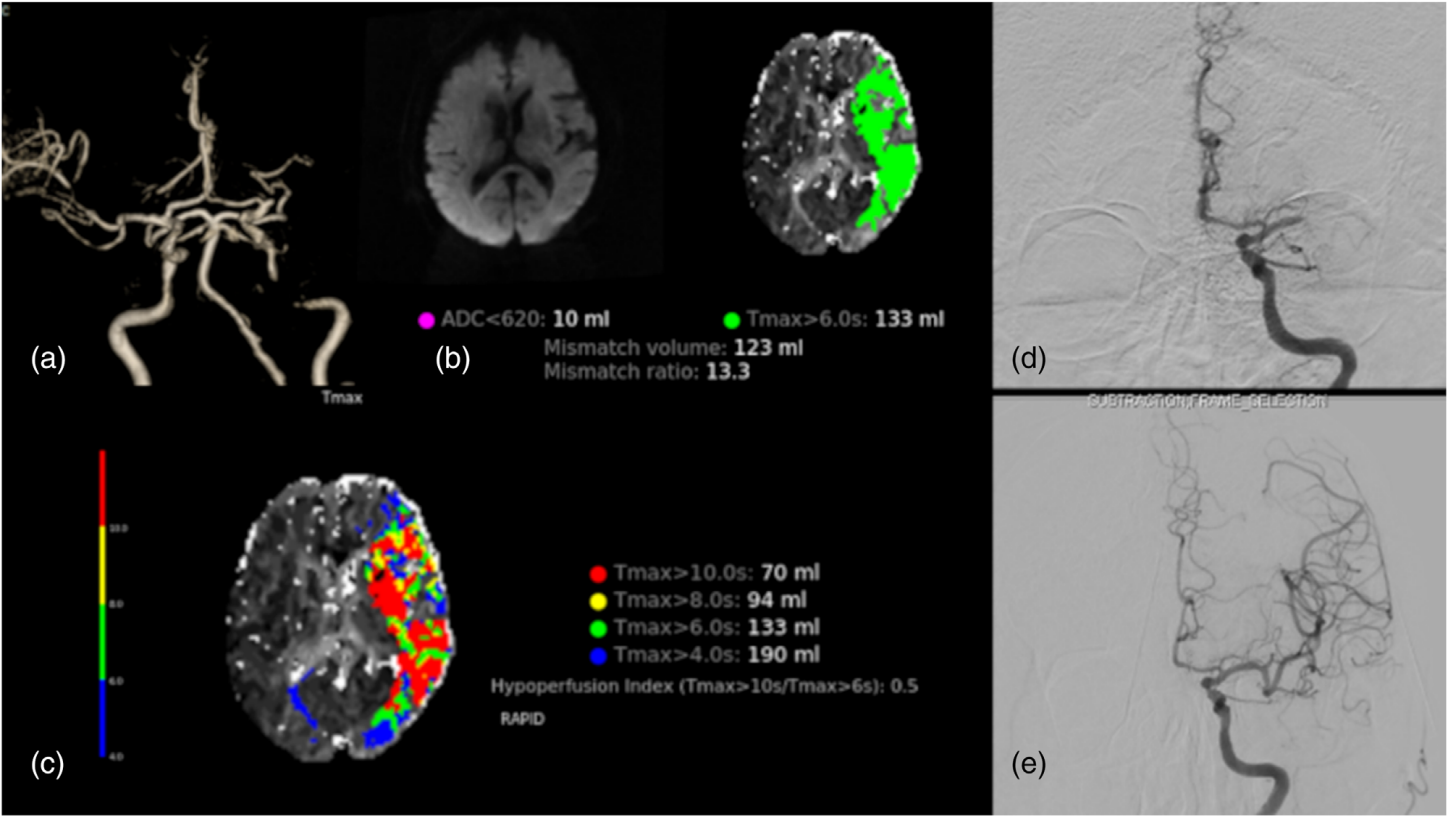
(A) MR angiography. (B) DWI‐PWI map. (C) *T*max map. (D) Angiography before endovascular treatment. (E) Angiography after endovascular treatment.

A patient in their 70s presented with hypertension and AF. NIHSS on arrival was 18, MRA showed occlusion in the M1 segment of the left MCA (Figure 4A), and MRI and MRP showed a core volume of 10 mL and a *T*max > 6 s volume of 133 mL. DWI‐PWI mismatch was positive. *T*max > 6 s volume/*T*max > 4 s volume was 0.7 and *T*max > 8 s/*T*max > 4 s was 0.49 (Figure 4C). Cerebral angiography showed occlusion of a left MCA, and recanalization was achieved (Figure 4D–E). This case was diagnosed as CE because no intracranial stenosis was seen after EVT.

#### 3.1.2 Case 2

**FIGURE 5:**
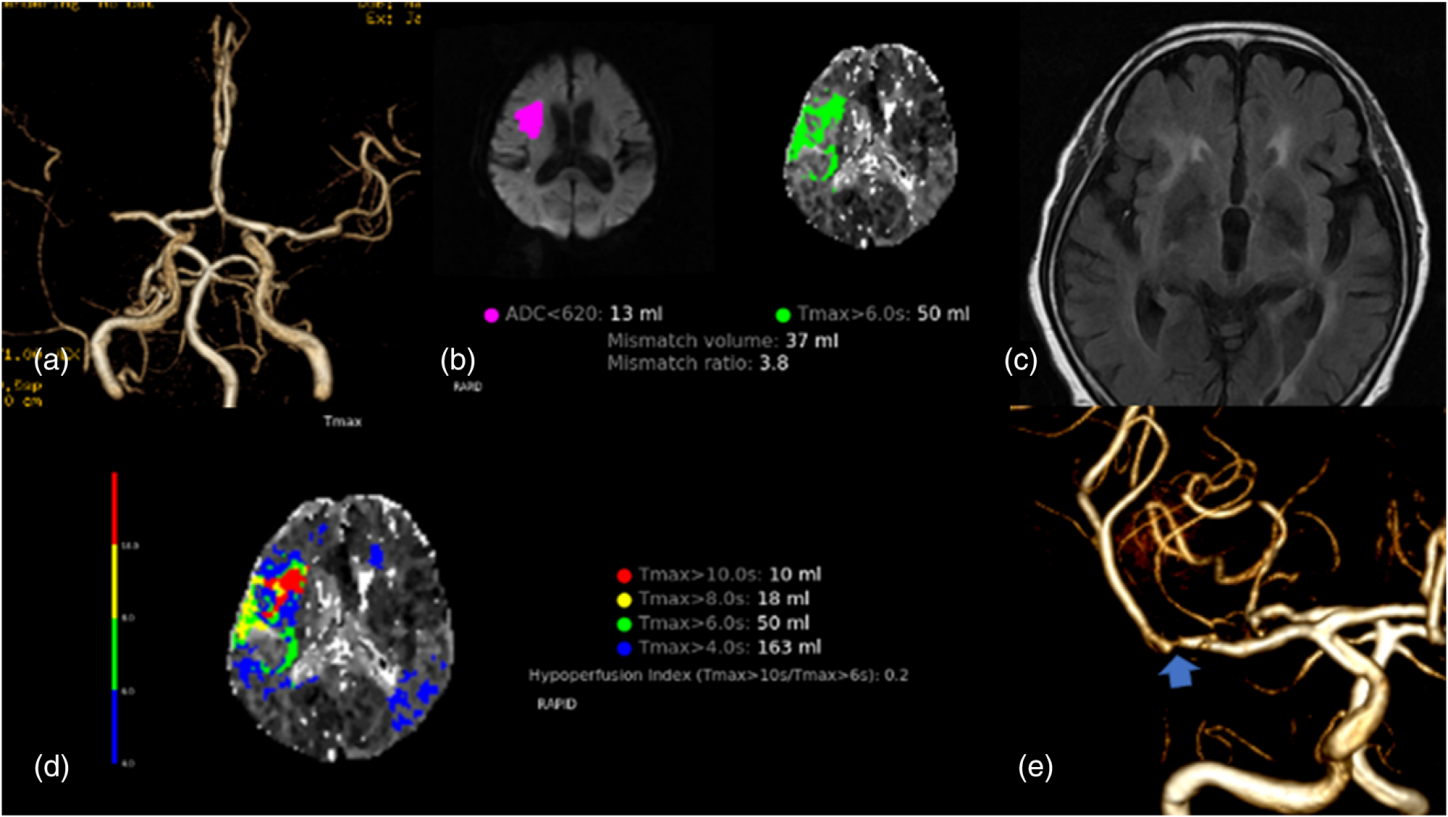
(A) MR angiography. (B) DWI‐PWI map. (E) FLAIR imaging. (D) *T*max map. (E) MR angiography before onset.

A patient in their 70s presented with hypertension, diabetes, and no AF. NIHSS on arrival was 6. MRA showed occlusion in the M1 segment of the right MCA (Figure 5A). MRI and MRP showed a core volume of 13 mL and a *T*max > 6 s volume of 50 mL. DWI‐PWI mismatch was positive (Figure 5B). Fluid‐attenuated inversion recovery (FLAIR) imaging showed hyperintense vessel sign in the M2 segment of the right MCA (Figure 5C). *T*max > 6 s volume/*T*max > 4 s volume was 0.31 and *T*max > 8 s/*T*max > 4 s was 0.11 (Figure 5D). MRA showed severe stenosis of the right MCA before onset (Figure 5E). This case was diagnosed as ICAS‐LVO.

## 4 DISCUSSION

The present study showed *T*max volumes and collateral scores were associated with clinical type. In particular, *T*max > 6 s volume/*T*max > 4 s volume was significantly higher in the CE group than in the ICAS‐LVO group, and high *T*max > 6 s volume/*T*max > 4 s volume showed an association of clinical type by multiple logistic regression analysis. These findings suggest that high *T*max > 6 s volume/*T*max > 4 s volume can predict collateral flow and clinical type. Prediction of clinical type prior to EVT by *T*max volume evaluation would allow not only more accurate determination of the indications for EVT, but also prompt decision‐making on the timing of antiplatelet administration and the choice of endovascular device for mechanical thrombectomy. We have performed perfusion imaging also for cases within 6 h of onset, and we think that it is worth to measure *T*max volume even if it takes time.

We focused on the difference of the collateral flow between CE and ICAS‐LVO. The present study showed that patients in the ICAS‐LVO group were more likely to have intermediate or excellent collateral flow, whereas those of CE group were more likely to have poor collateral flow.

Collateral flow is a pivotal component in the pathophysiology of intracranial atherosclerosis (Liebeskind et al., 2011). Secondary collateral pathways, such as leptomeningeal anastomoses, which require time to develop are presumed to be recruited once primary collaterals at the circle of Wills have failed (Liebeskind, 2003). The pathomechanism of ICAS‐LVO is likely due to in‐situ thromboocclusion rather than embolic occlusion (Kang et al., 2014). Given the longer opportunity for collateral development, ICAS‐LVO may more often present with fluctuating or low severity clinical symptoms (Lee et al., 2022). The incidence of slow progressors may be higher in ICAS‐LVO than in patients with other stroke subtypes (Suk Jae Kim et al., 2009). Patients with ICAS‐LVO may have different hemodynamic (collateral status) features on neuroimaging compared to patients with embolic LVO (Bang et al., 2019). LVO due to progressive atherosclerotic stenosis of an intracranial segment may allow robust collateral flow to develop over time, unlike the scenario accompanying CE‐LVO (Liebeskind et al., 2011).

Several studies have previously reported on clinical type diagnosis. Clinical history, including low median baseline NIHSS score, male sex, hypercholesterolemia, and smoking, may possibly support ICAS‐LVO, while AF suggests CE (Jin et al., 2020). Although AF is useful for clinical type diagnosis, it may not be recognized at the arrival. One study reported that BNP level was useful in distinguishing CE from non‐CE (Igarashi et al., 2019). Imaging features including no hyper‐dense vessel sign on noncontrast enhanced CT, no susceptibility vessel sign on susceptibility‐weighted MRI, truncal‐type occlusion on CTA, occlusion in the M1 segment of the MCA, scattered or borderzone infarct pattern on DWI, arterial calcification and tortuosity on the angiography, and residual arterial stenosis after EVT are considered suggestive of ICAS‐LVO (Park et al., 2019). A jet‐like appearance on angiography in 20.7% (34 patients) and noted that a jet‐like appearance offered an image marker for ICAS‐LVO in a retrospective study of 164 patients (Jin et al., 2020). However, the identification of clinical type before EVT by these imaging features remains challenging, particularly in patients with complicated clinical situations. We therefore focused on analysis using RAPID, which can provide data before EVT. Acute diagnostic imaging has become increasingly important with the expansion of eligibility criteria for EVT in AIS to tissue‐based selection and various imaging biomarkers for predicting symptom onset time have been reported in recent years (Albers et al., 2018; Kishi et al., 2022; Ma et al., 2019; Nogueira et al., 2018). DWI‐PWI mismatch or CBF‐PWI mismatch by the analysis of RAPID has been used to judge eligibility for EVT in AIS, including in patients with unknown symptom onset time, and has expanded the recommendations of eligibility for EVT (Albers et al., 2018). Perfusion imaging has become an essential tool for deciding between EVT and medical care in AIS (Demeestere et al., 2020; Laughlin et al., 2019).

During analysis by RAPID, *T*max volumes seemed to show different characteristics between the ICAS‐LVO group and CE group, so we focused on *T*max volumes in the present study. The present results revealed that patients in the CE group had a larger median *T*max volume and *T*max ratio than patients in the ICAS‐LVO group. The perfusion parameter *T*max > 6 s is often used to estimate tissue that is likely to progress to infarction if reperfusion is not achieved. Mismatch between core volume and *T*max > 6 s volume estimates salvageable tissue (Gregory W. Albers, 2018; Inoue, 2019). Several studies have reported on associations between *T*max volume and collateral flow. Galinovic et al. (2018) reported that CBF/*T*max > 4 s volume ratio and CBF/*T*max > 6 s volume ratio were significantly associated with poor collateral status in a retrospective study of 35 patients with AIS. Lyndon et al. (2021) reported that HIR, *T*max > 6 s volume, and *T*max > 10 s volume were associated with CTA collateral status in a retrospective study of 52 patients with LVO AIS. The present study showed that *T*max > 6 s volume/*T*max > 4 s volume was associated with the collateral score. *T*max volume and *T*max volume ratio, which were associated with collateral flow, could therefore predict clinical type.

Several studies have reported on associations between *T*max volume and clinical type. Patients with definitive AF had more severe hypoperfusion (median *T*max > 8 s volume 48 vs. 29 mL, *p* = .02) compared to patients with no AF, most likely attributable to poorer collateral circulation in a retrospective study of 175 patients with AIS (Tu et al., 2015). Suk Jae Kim et al. (2009) reported that intracranial large artery atherosclerotic stroke had lower half of severity of perfusion defect (*T*max > 8 s volume/*T*max > 2 s volume) compared with other stroke mechanisms (odds ratio, 6.21; 95%CI, 1.14−33.74; *p* = .034). Kim et al. (2021) reported that ICAS infarct patterns may correlate with distinct perfusion profiles in a study of 42 patients with AIS due to subocclusive (50−99%) intracranial stenosis. According to that report, median *T*max > 4 s volume/*T*max > 6 s volume was 7 in branch occlusive disease, 3.7 in thromboembolic ischemia, and 4.6 in internal borderzone ischemia.

Haussen et al. (2018) reported an automated CTP *T*max > 4 s volume/*T*max > 6 s volume ratio ≥ 2 was independently associated with ICAD‐LVO in a retrospective study of 250 patients with intracranial atherosclerotic disease (ICAD; n = 21) and non‐ICAD (n = 229) etiologies with AIS undergoing EVT. They suggested that an automated CTP profile exhibiting a disproportionately large volume of *T*max > 4 s tissues relative to *T*max > 6 s volume may favor the presence of an LVO with underlying ICAD. In the present study, *T*max > 6 s volume/*T*max > 4 s volume (*T*max > 4 s volume/*T*max > 6 s volume) was 0.59 (1.69) in the CE group and 0.40 (2.50) in the ICAS‐LVO group on CTP/MRP. The critical *T*max > 6 s volume/*T*max > 4 s volume (*T*max > 4 s volume/*T*max > 6 s volume) cutoff for predicting clinical diagnosis was determined to be 0.5 (2.0). These results were consistent with findings from previous reports.

Although absolute rates of successful reperfusion were similar between IVAS‐LVO and CE groups, patients with ICAS‐LVO showed relatively poor functional outcomes compared to those with CE. The relatively poor outcome in the ICAS‐LVO group is mainly attributable to a longer procedure time, reflecting procedural complexity, and a higher rate of reocclusion (Lee et al., 2018). Rescue treatments, including balloon angioplasty, rescue stenting, and intra‐arterial glycoprotein IIb/IIIa inhibitor infusion, can be considered for ICAS‐LVO refractory to stent retriever (Baek et al., 2018; Park et al., 2019). Accurate early diagnosis of ICAS‐LVO prior to EVT may overcome those causes of poor ICAS‐LVO outcomes and improve clinical prognosis for patients with ICAS‐LVO. *T*max volumes and *T*max > 6 s volume/*T*max > 4 s volume obtained from perfusion imaging may be useful for clinical type diagnosis before EVT.

### 4.1 Limitations

Several limitations to the present study should be considered. First, *T*max > 6 s volume/*T*max > 4 s volume cannot evaluate Tmax volume. If *T*max > 4 s volume is too small, *T*max > 6 s volume/*T*max > 4 s volume may become abnormally high. In the present study, no concerns were seen for evaluation because median *T*max > 4 s volume was 234 mL and *T*max > 6 s volume/*T*max > 4 s volume was larger in the CE group than in the ICAS‐LVO group for every location of occlusion. Second, we could not perform cerebral angiography for 14 patients. Chronic occlusion and severe stenosis cases may be included in the ICAS‐LVO groups. However, we only included cases in which intracranial atherosclerosis clearly led to acute occlusion by evaluating previous imaging for patients with no cerebral angiography. Third, collateral flow may not always be associated with clinical type. Even in ICAS‐LVO cases, collateral flow may not be well developed. In fact, *T*max > 6 s volume/*T*max > 4 s volume was high in one middle‐aged male in the ICAS‐LVO group who may not have developed collateral flow in the present study. In present small number of cases, there was a significant difference between the collateral score and *T*max > 6 s volume/*T*max > 4 s volume, but accumulation of cases is necessary. Fourth, this study was retrospective in nature and all study subjects were drawn from a single stroke center, limiting the generalizability of the study results. Fifth, the sample size was small. Our results should be validated in a larger patient sample in future studies. Despite these limitations, *T*max volumes and *T*max > 6 s volume/*T*max > 4 s volume can be calculated quickly and easily on MRP/CTP before EVT in emergent stroke situations. Our findings suggest that *T*max volumes and *T*max > 6 s volume/*T*max > 4 s volume have value for estimating the clinical type. Since RAPID is an analysis software for anterior circulation occlusion, only anterior circulation occlusion was targeted in this study. However, distinguishing ATBI from CE is an important issue even for posterior circulation occlusion such as basilar artery occlusion. In the future, we plan to verify whether *T*max volume can predict the clinical type of posterior circulatory occlusion.

## 5 CONCLUSIONS

*T*max volumes and *T*max > 6 s volume/*T*max > 4 s volume obtained from perfusion imaging can predict clinical type in patients with AIS prior to EVT.

## AUTHOR CONTRIBUTIONS

IN and FK conceived of the study, analyzed and interpreted the data, drafted the manuscript, and edited the manuscript. All authors have reviewed and approved the final version of the manuscript.

## FUNDING

This work did not receive any grants from funding agencies in the public, commercial, or not‐for‐profit sectors.

## CONFLICT OF INTEREST STATEMENT

The authors declare no conflict of interest.

### PEER REVIEW

The peer review history for this article is available at https://publons.com/publon/10.1002/brb3.3163.

## Data Availability

Data are available upon reasonable request.
